# Autophagy Disruptions Associated With Altered Optineurin Expression in Extranigral Regions in a Rotenone Model of Parkinson's Disease

**DOI:** 10.3389/fnins.2018.00289

**Published:** 2018-05-16

**Authors:** John P. Wise, Charles G. Price, Joseph A. Amaro, Jason R. Cannon

**Affiliations:** ^1^School of Health Sciences, Purdue University, West Lafayette, IN, United States; ^2^Purdue Institute for Integrative Neuroscience, Purdue University, West Lafayette, IN, United States

**Keywords:** Parkinson's disease, rotenone, autophagy, optineurin, LC3

## Abstract

The motor features of Parkinson's disease (PD) primarily result from a lesion to the nigrostriatal dopamine system. Numerous non-motor symptoms occur in PD, many of which are postulated to stem from pathology outside of the nigrostriatal dopamine system. Perturbations to protein trafficking, disruption of mitochondrial integrity, and impaired autophagy have repeatedly been implicated in dopaminergic neuron cell death. Previously, we demonstrated that multiple markers of autophagy are disrupted in a rotenone model of PD, with alterations occurring prior to an overt lesion to the nigrostriatal dopamine system. Whether these events occur in extra-nigral nuclei in PD and when relative to a lesion in the nigrostriatal dopamine system are generally unknown. The primary goal of these studies was to determine whether autophagy disruptions, in non-dopaminergic neuronal populations occur in an environmental model of PD utilizing a mitochondrial toxin. Here, we utilized the rat rotenone PD model, with sampling time-points before and after an overt lesion to the nigrostriatal dopamine system. In analyzing autophagy changes, we focused on optineurin (OPTN) and the autophagy marker, LC3. OPTN is an autophagy cargo adapter protein genetically linked to amyotrophic lateral sclerosis and glaucoma. In the present study, we observed OPTN enrichment in all PD-relevant brain regions examined. Further, alterations in OPTN and LC3 expression and colocalized puncta suggest specific impairments to autophagy that will inform future mechanistic studies. Thus, our data suggest that autophagy disruptions may be critical to PD pathogenesis in non-dopaminergic neurons and the onset of non-motor symptoms.

## Introduction

Parkinson's disease is a progressive debilitating neurological disease, where only a small proportion of cases have a known genetic link (<10%). The vast majority of cases have an unknown sporadic incidence which are thought to be caused by gene-environment interactions (McCulloch et al., 2008; Gao and Hong, 2011; Myers et al., 2013). Currently, clinical diagnosis relies on the evidence of several motor symptoms with attenuation by levodopa treatment; including a resting tremor, bradykinesia, stooped posture, and increased muscle tone, among others (Postuma et al., 2015). While motor symptoms primarily result from the loss of dopaminergic neurons and signaling from the nigrostriatal dopamine system, non-motor deficits are thought to arise from pathology to several other brain regions and the peripheral nervous system (Wolters, 2009). Non-motor symptoms often precede a clinical diagnosis by years to decades and are often just as debilitating as the motor symptoms. Prominent non-motor symptoms may include REM sleep disorder, olfactory dysfunction, delayed gastrointestinal motility (typically resulting in constipation), and some visual dysfunctions (e.g., blurred vision, diplopia; Schapira et al., 2017). PD is characterized by the loss of dopaminergic neurons in the substantia nigra pars compacta, with ~60% of this population lost by the time of clinical diagnosis (Bernheimer et al., 1973; Gelb et al., 1999).

A significant amount of PD research has focused on why the dopaminergic neurons of the substantia nigra pars compacta are selectively vulnerable. For example, dopaminergic neurons of the immediately adjacent ventral tegmental area are relatively spared and neurodegeneration is less severe in other affected regions (McRitchie et al., 1997; Braak and Braak, 2000). Much of the research points to the energetic stress of the nigral dopamine neurons; these neurons exhibit poorly myelinated axons, large dendritic processes, and a limited mitochondrial population (Double, 2012; Brichta and Greengard, 2014; Sanders et al., 2014a; Haddad and Nakamura, 2015; Pacelli et al., 2015). Further research in these neurons has revealed multiple coalescent dysfunctional pathways that contribute to their deterioration, including but not limited to; impairments in vesicle trafficking, iron accumulation, impaired oxidative stress management, mitochondrial dysfunction, Golgi fragmentation, misfolded and aggregated proteins, and impaired pathways for protein clearance (Bindoff et al., 1989; Anglade et al., 1997; Baba et al., 1998; Fujita et al., 2006; Hauser and Hastings, 2013; Hwang, 2013). However, relatively little research has considered such dysfunction in the extranigral regions that are affected. Clearly, many other brain regions are important in PD. For example, Braak et al. have demonstrated Lewy body pathology (LBP) in many extranigral regions and have proposed this pathology progresses up the brainstem, starting in the dorsal motor vagal nucleus and reaching the entire neocortex (Braak et al., 2003). Importantly, many of these regions affected are closely linked to nonmotor symptoms associated with PD and some regions also show signs of neurodegeneration (Braak et al., 1996; Wolters, 2009).

**Figure 1:**
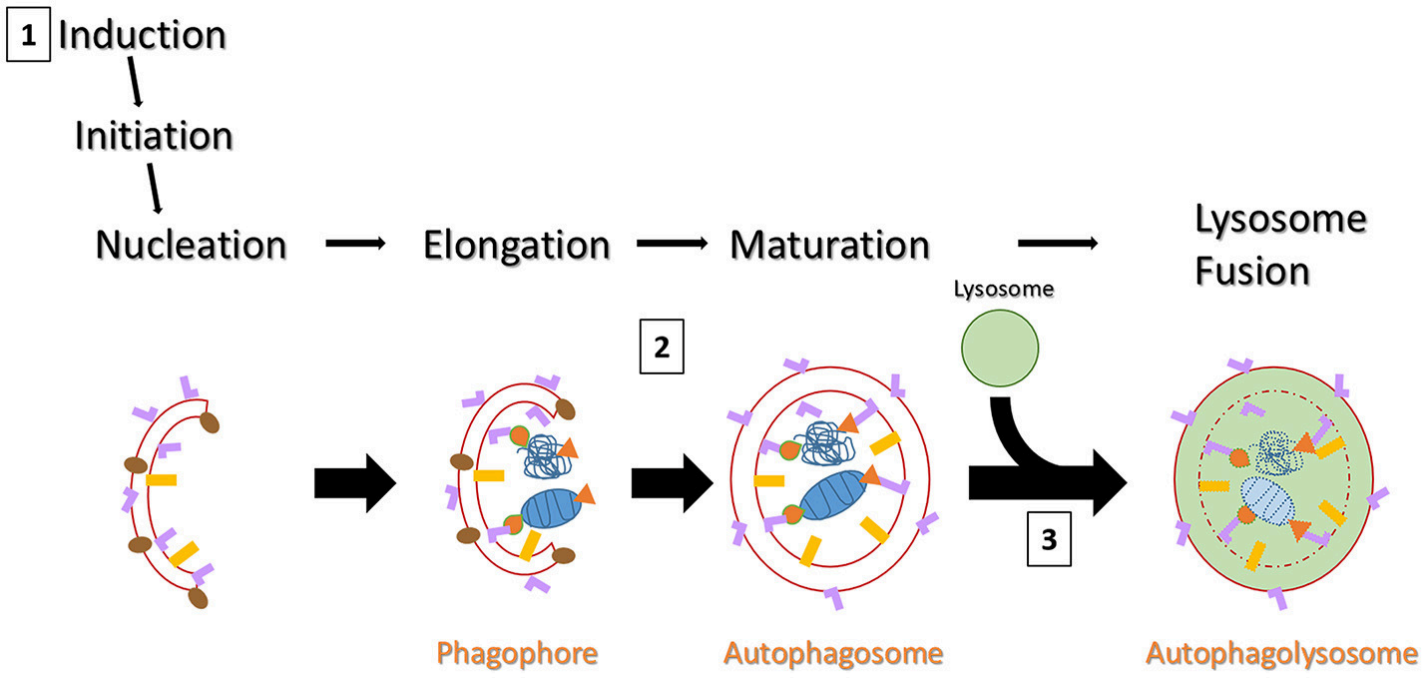
Schematic overview of autophagy. Autophagy can be induced by a chemical stimulus or by amino acid starvation. Upon induction of autophagy, LC3-I is cleaved into LC3-II (pink “L”) and an LC3-II positive membrane structure begins to form (usually on or near the endoplasmic reticulum). Meanwhile, GABARAP (gold rectangles) is expressed on the pre-autophagosomal structure during nucleation. GABARAP serves to recruit ULK1 (not pictured) and Beclin-1 (brown ovals). Beclin-1 serves as a scaffold protein to recruit more autophagy proteins and plays a key role in regulating autophagosome development. GABARAP then serves a similar role as LC3-II for cargo recruitment, but interacts with different cargo adaptors (not pictured) than LC3-II. As the membrane grows, various cargo are brought to the interior via cargo adaptor proteins (e.g., p62 or OPTN). Here, cargo is represented by a mitochondrion and a protein aggregate (both in blue) with orange p62 (triangles) and OPTN (tear drops) acting as cargo adaptors. The membranes fuse, forming a double-membraned autophagosome, marked by LC3-II on both inner and outer fuses with a lysosome and begins membranes. After maturity, the autophagosome lysosomal degradation of the inner membrane and all its constituents. There are three likely points of dysfunction that could result in detectable pathology *in vivo*: **(1)** failed induction would results in fewer autophagosomes being formed but little to no impact on expression and puncta formation of cargo adaptor proteins; **(2)** failed or delayed binding of LC3-II with cargo adaptor proteins would result in increased LC3 and cargo adaptor puncta, but decreased colocalized puncta; **(3)** failed lysosome fusion would result in all puncta being increased.

Autophagy is a catabolic process that serves as a clearance pathway for protein aggregates, cytoplasmic components, and subcellular organelles (e.g., mitochondria; Glick et al., 2010). Figure 1 provides a schematic overview of the autophagy pathway, with critical points of dysfunction numbered for discussion. Beclin-1 interacts with ULK1 to induce autophagy. Specifically, beclin-1 is important for recruiting autophagy proteins to the pre-autophagosomal structure, during nucleation (Kang et al., 2011). Furthermore, GABARAP serves as a scaffolding protein to recruit ULK1 and beclin-1 to the site of nucleation. Upon induction of autophagy, microtubule associated protein light chain I (LC3-I) is cleaved into LC3-II and initiates the formation of a phagophore. The phagophore enters an elongation phase as cargo are recruited for degradation via cargo adaptor proteins (e.g., p62, optineurin). LC3 is the most studied autophagy protein and has been shown to interact with a variety of cargo adaptor proteins (e.g., p62, OPTN, FYCO1, NBR1). GABARAP also interacts with various cargo adaptors for cargo recruitment to the autophagosome during elongation (Schaaf et al., 2016). During elongation, GABARAP interacts with the cargo adaptors ALFY or NBR1 to recruit dysfunctional mitochondria and interacts with FYCO1 to recruit protein aggregates to the developing autophagosome. Due to this apparent overlap in function, LC3 and GABARAP have been studied to understand their critical functions in autophagy. Cells deficient in either protein show impaired autophagosome formation; GABARAP deficiency results in larger autophagosomes, while LC3 deficiency results in smaller autophagosomes. The membrane then fuses to form a mature autophagosome (a double-membraned structure marked by LC3-II on the inner and outer membranes). Finally, the mature autophagosome fuses with a lysosome, resulting in the lysosomal degradation of the inner membrane and the enclosed cargo (Klionsky et al., 2016). Autophagy is known to be impaired in PD in postmortem patient brains, in genetic and environmental *in vivo* and *in vitro* models, and has been suggested as a peripheral biomarker of PD (Anglade et al., 1997; Zhu et al., 2003; Higashi et al., 2011). However, whether such impairment occurs outside the substantia nigra is poorly understood. Currently it is unclear exactly how autophagy is impaired. Here, literature from various models points to impairments in induction, cargo recruitment, and autophagosome-lysosome fusion (Schapira, 2008; Sanchez-Perez et al., 2012). In support of the critical importance for autophagy in PD, a recent paper demonstrated PD-like behavior and pathology in the brains of mice with autophagy deficiency specific to dopaminergic neurons (Sato et al., 2018). Here, the authors reported significantly increased foot slips in autophagy-deficient mice on a runway test, deposition of alpha-synuclein aggregates in dopaminergic neurites, aggregates immuno-positive for p62 and ubiquitin in these neurons, decreased TH^+^ neurons in the SNpc, and decreased dopamine and dopamine metabolites in the dorsal striatum.

OPTN is a cytoplasmic protein that serves many different roles in the cell (Ying and Yue, 2012; Slowicka et al., 2016). OPTN mutations are linked to amyotrophic lateral sclerosis, frontotemporal lobar degeneration, and normal-tension glaucoma; but it is also observed in protein inclusions present in Alzheimer's disease, Huntington's disease, and PD (Osawa et al., 2011; Ying and Yue, 2012). One of the most well studied roles of OPTN is its function as a cargo adaptor in autophagy; OPTN exhibits an LC3 interacting region near its N-terminus, and cargo recognition domains at its C-terminus (ubiquitin binding domain and coiled-coils) (Korac et al., 2013; Rogov et al., 2013; Ying and Yue, 2016). Point mutations and OPTN truncations impair its cargo adaptor function, impairing autophagy and contributing to cell death. Intriguingly, the glaucomatic OPTN^M98K^ mutation was found to be a risk factor for PD in a recent genome wide association study, and PD patients exhibit a higher prevalence of developing glaucoma (Lill et al., 2012; Nucci et al., 2015; Sirohi et al., 2015). We previously demonstrated OPTN is robustly expressed in nigral dopamine neurons, and its expression and interaction with LC3 is elevated after rotenone exposure in rats (Wise and Cannon, 2016). However, expression in other brain nuclei and changes in such expression in PD models are unknown. Our aim here was to address the key gaps in the literature with respect to brain-wide autophagy changes through testing the hypothesis that autophagy disruption would occur in extra-nigral nuclei important in PD and that such changes would precede a lesion to the nigrostriatal dopamine system.

## Materials and methods

### Overall experimental design relative to previous studies

Detailed methodology is described below. However, it is worth noting that the exact animals utilized in this study have been well-characterized for lesion development in the nigrostriatal dopamine system and autophagic disruption in dopaminergic neurons (Wise and Cannon, 2016). None of the analyses in that publication overlap with this report, which has a different set of experimental goals. The main goal here was to examine autophagic disruptions in brain nuclei linked to non-motor PD symptoms in response to treatment with rotenone, a known mitochondrial toxin used to model PD. With the temporal development of the lesion already published in these animals and also extensively characterized in other studies (Cannon et al., 2009; Wise and Cannon, 2016), we are able to determine if changes in other brain regions precede an overt lesion to the nigrostriatal dopamine system.

### Animals

All animals were male wild-type Lewis rats purchased from Harlan (now Envigo; Indianapolis, IN) and were between the ages of 31–42 weeks when euthanized. Rats were housed under standard 12 h light cycle, with free access to water and fed *ad libitum*. This study was carried out in accordance with the recommendations of United States Department of Agriculture (USDA) and the United States Public Health Service (USPHS) in accordance with the Animal Welfare Act and Purdue's Animal Welfare Assurance The protocol was approved by the Purdue University Animal Care and Use Committee.

### Chemicals and reagents

Primary antibodies for rabbit anti-OPTN (ab23666) and rabbit anti-beclin-1 (ab62557) were purchased from abcam (Cambridge, MA, USA); mouse anti-LC3 was purchased from MBL International Corporation (M152-3, Woburn, MA, USA); mouse anti-alpha-synuclein was purchased from BD Transduction Labs (610786, San Jose, CA, USA); chicken anti-MAP2 (AB5543), sheep anti-tyrosine hydroxylase (AB1542), anti-tryptophan hydroxylase (AB1541), and anti-choline acetyltransferase (AB1582) were purchased from EMD Millipore (Billerica, MA, USA). Normal donkey serum (017-000-121) and secondary antibodies, Alexa Fluor^®;^ 647 anti-sheep (713-605-147), Alexa Fluor^®;^ 488 anti-rabbit (711-545-152), and Cy^TM^3 anti-mouse (715-165-151) were purchased from Jackson ImmunoResearch Laboratories, Inc. (West Grove, PA, USA). Rotenone (R8875) and glycerol (G5516) were purchased from Sigma-Aldrich (St. Louis, MO, USA). Triton X-100 was purchased from Fisher BioReagents (BP151-500, Fair Lawn, NJ).

### Rotenone administration

Rotenone was administered at 3.0 mg/kg/day by intraperitoneal injection as previously described (Cannon et al., 2009). This model has been extensively characterized in terms of the temporal development of behavioral, neurochemical, and neuropathological alterations in the nigrostriatal dopamine system that are relevant to PD (Cannon et al., 2009, 2013; Cannon and Greenamyre, 2010; Tapias et al., 2010, 2014; Zharikov et al., 2015). Of note, the dose and/or time of exposure can be adjusted to produce a preclinical model, where early-stage pathogenesis can be studied, prior to overt cell death (Drolet et al., 2009; Sanders et al., 2014a,b). In the present study, we chose to sample animals at preclinical time-points, where overt motor phenotypes were not yet present and at end-stage, where postural instability, rigidity, and bradykinesia were present. These deficits have been well described in the rotenone model at this dose (Cannon et al., 2009). Thus, animals were euthanized after 24 hours (*n* = 3), 5 days (*n* = 3), or at end-stage (*n* = 3) (typically at 9–12 days). In this model, overt behavioral deficits are associated with a lesion to the nigrostriatal dopamine system (Betarbet et al., 2000; Cannon et al., 2009), while sampling at 5 days or before is prior to the development of an detectable lesion (Sanders et al., 2014a,b). Of note, the temporal development of a nigrostriatal dopamine system has been characterized in the animals used in this report (Wise and Cannon, 2016).

### Brain regions analyzed and markers chosen for analysis

We analyzed changes to expression and punctate forms of OPTN and LC3 in four nuclei implicated in Braak's hypothesis of PD: the dorsal motor vagal nucleus (10N), magnocellular raphe (RMg), locus coeruleus (LC), pedunculopontine tegmentum nucleus (PTg). We also considered changes to expression of alpha-synuclein and beclin-1 in the substantia nigra pars compacta (SNpc) because we previously showed that OPTN and LC3 expression and puncta were altered in this brain region (Wise and Cannon, 2016). These regions were chosen because they are implicated in Braak's hypothesis of Lewy body progression in PD and they are linked to many PD nonmotor symptoms (Braak et al., 2003; Wolters, 2009). For example, constipation and REM sleep behavior disorder have been linked to pathology in the 10N and the LC, respectively (Cersosimo and Benarroch, 2012; García-Lorenzo et al., 2013). Specific details of processing and analyses are given below.

### Immunohistochemistry

Rats were placed under deep anesthesia with pentobarbital (>50 mg/kg) (Beuthanasia-D Special, Schering-Plow Animal Health Corp, Union, NJ, USA), then transcardially perfused with 100–150 mL PBS, followed by 250–300 mL 4% buffered paraformaldehyde (PFA). Brains were surgically removed, post-fixed in 4% PFA for ~24 h, and then saturated with 30% sucrose at 4°C for at least 5–7 days until sinking. Each brain was coronally sectioned on a frozen sliding microtome (Microm HM 450, Thermo Scientific) at a 35 μm thickness and stored in cryoprotectant at −20°C until used for staining. Brain sections containing the desired regions were randomly selected, rinsed in 10x PBS for 10 min, six times, at room temperature (RT) on an open-air platform shaker; blocked in 10% normal donkey serum (NDS, cat. # 017-000-121) in PBS with 0.3% Triton X-100 (PBS-T) for 90 min at RT; incubated with primary antibodies for ~48 h in PBS-T with 1% NDS at 4°C. The sections were then rinsed three times with 10x PBS at RT for 10 min each time; incubated with secondary antibodies in PBS-T with 1% NDS for 90 min at RT; and rinsed six times with 10x PBS at RT for 10 min each time before being mounted on slides and coverslipped as a wet mount using 50/50 glycerol:PBS solution. Primary antibody dilutions were: mouse anti-LC3 (1:1000); rabbit anti-OPTN (1:5000); sheep anti-tyrosine hydroxylase (1:1000); sheep anti-tryptophan hydroxylase (1:4000); sheep anti-choline acetyltransferase (1:1500). Secondary antibody dilutions were: Alexa Fluor^®;^ 647 anti-sheep, Alexa Fluor^®;^ 488 anti-rabbit, or Cy^TM^3 anti-mouse (1:500). In pilot studies, we optimized antibody titers using graded dilutions and columetric staining; then we performed staining for MAP2A (a pan-neuronal marker) + staining for autophagy markers to verify that the majority of total puncta were observed within neurons and not extracellular (we expect the few outside the MAP2A stain were present in glia).

### Microscopy and image analysis

Confocal images were captured on an inverted Nikon Eclipse TE 2000-U microscope with an EZ-C1 confocal, equipped with 10x, 20x, and 60x Plan Fluor objectives. Region of interest (ROI) analysis was then conducted on images using the NIS-Elements software ver. 4.30 to measure signal intensity. High magnification images (60x) were analyzed for puncta analysis; puncta were considered bright circular dots with diameters ranging from 0.5 to 2.0 μm, as has been previously described for autophagosome diameters (Mizushima et al., 2002). Changes in the % of colocalized puncta determined as # colocalized/#OPTN or # colocalized/#LC3; and were interpreted as described in Table 1, with the rationale for such conclusions addressed in the Discussion.

**Table 1:** Assessment of autophagy function or dysfunction by immunohistochemistry.

	**Expected outcome**		
**Expression data**	**LC3**	**OPTN**	
Induced, flux not impaired	↑	↓	
Impaired induction	↓	↓	
Impaired interaction	↓	↑	
Failed autophagosome-lysosome fusion	↑	↑	
OPTN failure or exhaustion	Unknown	↓	
Autophagosome failure or exhaustion	↓	↑	
**Puncta data**	**LC3**	**OPTN**	Colocalized
Induced, flux not impaired	↑	↓ or no change	No change
Impaired induction	↓	↓ or no change	↓
Impaired interaction	↓ or no change	↑	↓
Failed autophagosome-lysosome fusion	↑	↑	↑
OPTN failure or exhaustion	Unknown	↓	↓
Autophagosome failure or exhaustion	↓	↑	↓
***%*** **Colocalized data**	**LC3**	**OPTN**	
Induced, flux not impaired	No change	No change	
Impaired induction	No change	↓	
Impaired interaction	↓	↓	
Failed autophagosome-lysosome fusion	↑	↑	
OPTN failure or exhaustion	↓	↑	
Autophagosome failure or exhaustion	↑	↓	

*The rationale for these conclusions is addressed in the Discussion*.

### Statistical analysis

Statistical analysis was conducted using GraphPad PRISM, ver. 6. Data for intensity and puncta analyses were found not to have a Gaussian distribution by the D'Agostino & Pearson omnibus normality test. Thus, nonparametric analysis was conducted using Kruskal-Wallis nonparametric ANOVA, followed by *post-hoc* analysis using the Dunn's test. *p* < 0.05 deemed significant for all tests.

## Results

### Dorsal motor vagal nucleus (10N)

**Figure 2:**
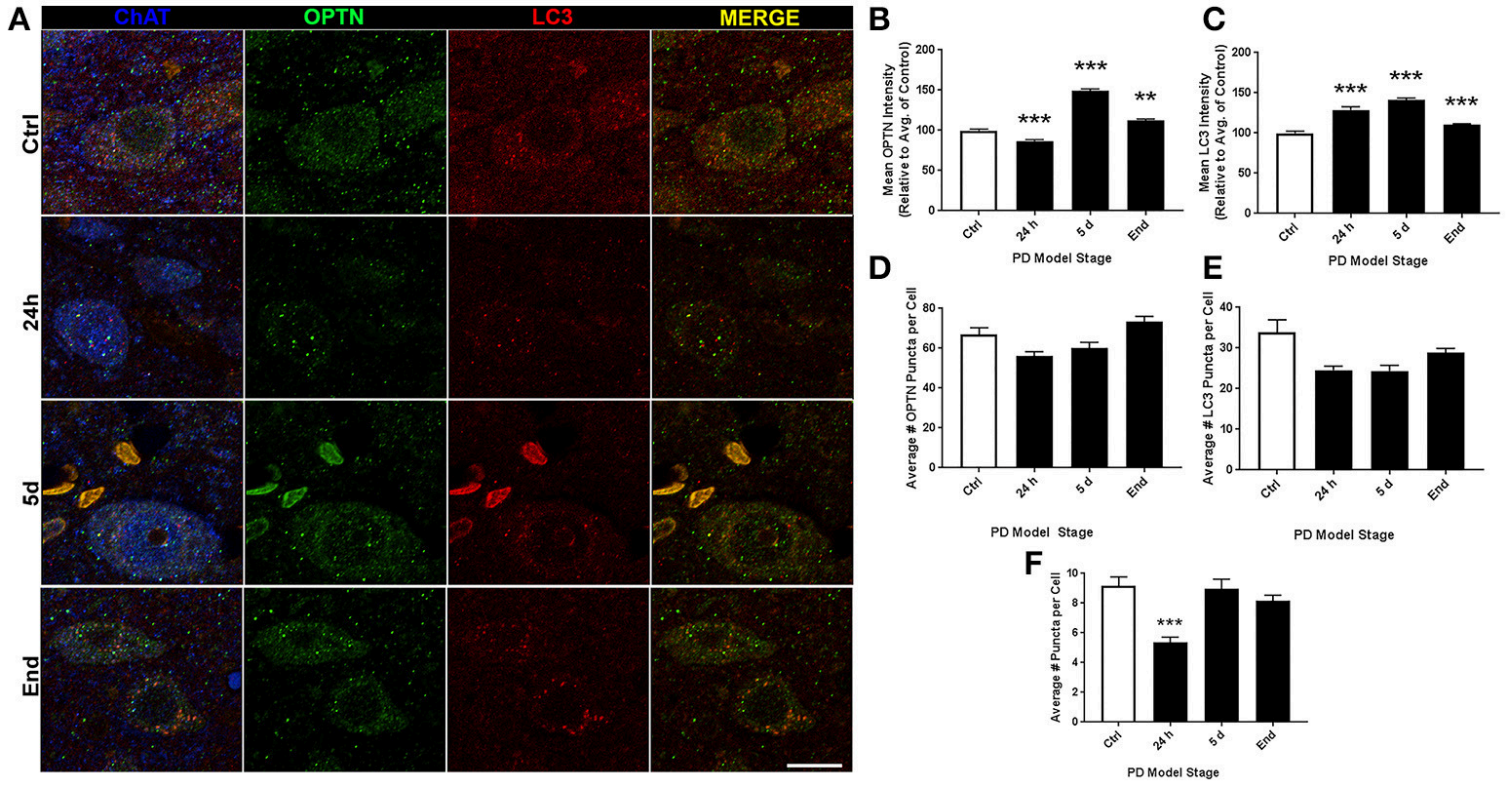
10N altered expression and puncta formation of OPTN and LC3 across PD stages. **(A)** Representative images of vagal cholinergic neurons co-stained with OPTN, LC3 and ChAT from animals treated as control(top), 24 h (second from top), 5 d (second from bottom), or end-stage (bottom); ChAT in blue, OPTN in green, LC3 in red (scale bar = 10 μm). **(B,C)** Quantitative analysis of OPTN and LC3 expression, respectively, measured by relative intensity of total cellular expression (relative to control); **(D–F)** mean number of puncta per neuron for OPTN, LC3 or colocalized puncta, respectively, across stages; data are presented as mean ± SEM; ^**^p < 0.01, ^***^p < 0.001 from control, Dunn's multiple comparison test after significant Kruskal-Wallis test.

ROI analysis was used to evaluate changes in OPTN and LC3 expression across stages of PD in our rat models: control (145 neurons), 24 h (123 neurons), 5 d (91 neurons), and end-stage (186 neurons); representative images are shown in Figure 2A. Figures 2B,C show changes in OPTN and LC3 expression; mean OPTN expression (relative to control) exhibited significantly decreased expression after 24 h rotenone (86.73 ± 1.615% of control, *p* < 0.001), while 5 d and end-stage expression were significantly increased (149.02 ± 2.53% and 112.33 ± 1.79%, respectively; *p* < 0.001 and < 0.01, respectively). Mean LC3 expression (relative to control) was significantly increased across all stages (128.89 ± 3.82%, 141.12 ± 2.39%, and 110.09 ± 1.50%, respectively; *p* < 0.001 in all stages). Punctate expression of OPTN, LC3 and colocalized puncta were also quantified; overtly identifiable puncta with a diameter of 0.5-2 um were counted. Figures 2D–F show the mean number of puncta per cell for OPTN, LC3, and colocalized puncta. There were no statistically significant differences detected across stages for OPTN or LC3 puncta. However, a statistically significant decrease in colocalized puncta was detected after 24 h rotenone. Here, the control mean was 9.2 ± 0.61 puncta per cell and 24 h mean was 5.4 ± 0.36 puncta per cell (*p* < 0.001). We then considered changes in the percent of OPTN or LC3 puncta that were colocalized by PD stage. Results show decreased percent of OPTN puncta and LC3 puncta were found colocalized only after 24 h rotenone when compared to control; 13.7 ± 0.8% and 10.8 ± 0.9% for OPTN (*p* < 0.01), 33.5 ± 2.0% and 23.1 ± 1.7% for LC3 (*p* < 0.001), respectively (**Figure 6A,B**).

### Magnocellular raphe (RMg)

**Figure 3:**
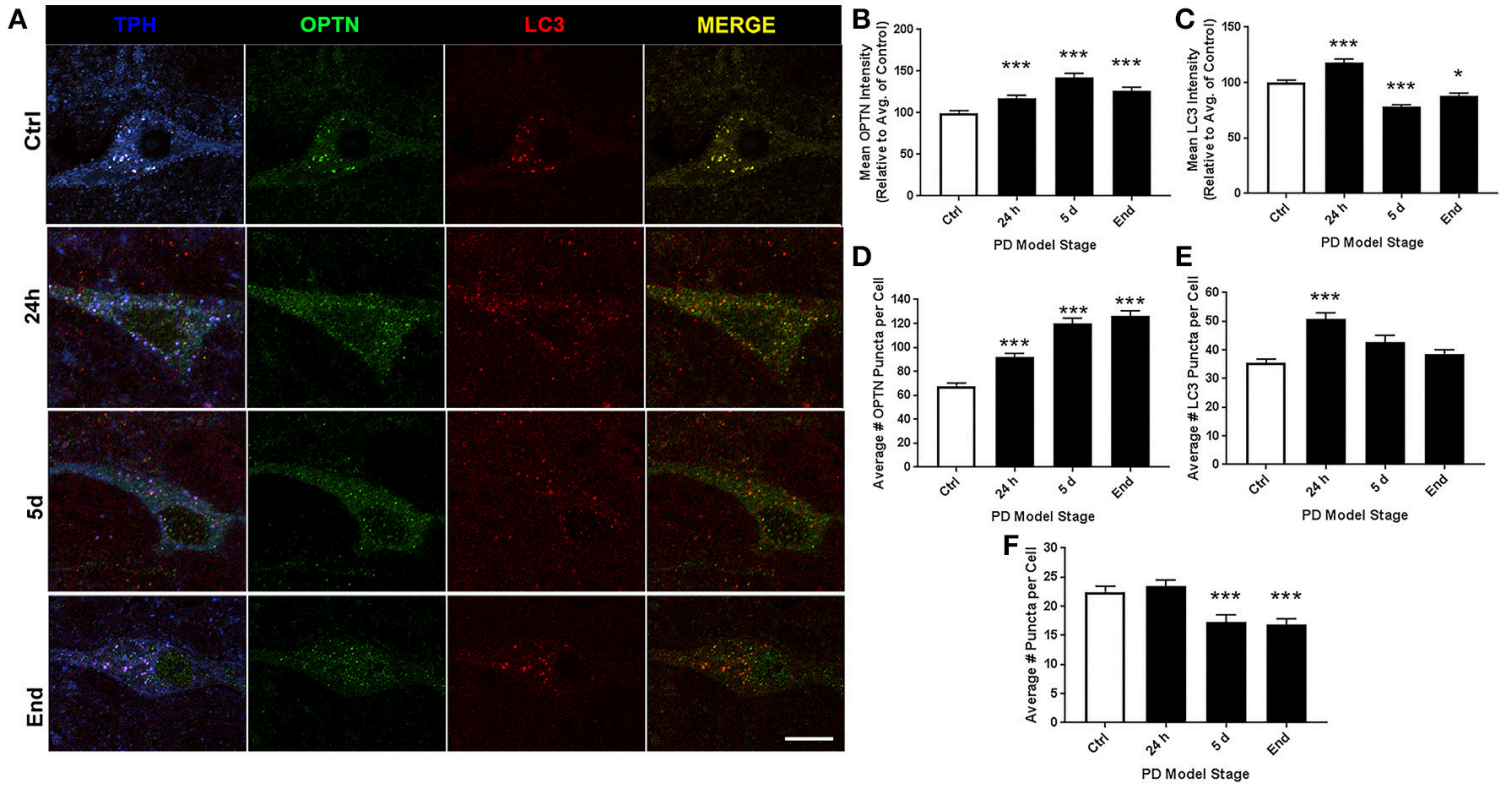
RMg altered expression and puncta formation of OPTN and LC3 across PD stages. **(A)** Representative images of raphe serotonergic neurons co-stained with OPTN, LC3 and TPH from animals treated as control(top), 24 h (second from top), 5 d (second from bottom), or end-stage (bottom); ChAT in blue, OPTN in green, LC3 in red (scale bar = 10 μm). **(B,C)** Quantitative analysis of OPTN and LC3 expression, respectively, measured by relative intensity of total cellular expression (relative to control); **(D–F)** mean number of puncta per neuron for OPTN, LC3 or colocalized puncta, respectively, across stages; data are presented as mean ± SEM; ^*^p < 0.05, ^***^p < 0.001 from control, Dunn's multiple comparison test after significant Kruskal-Wallis test.

Representative images of RMg staining are shown in Figure 3A. For ROI analyses we evaluated 161, 153, 124, and 110 neurons for control, 24 h, 5 d, and end-stage rats, respectively. Figure 3B shows mean OPTN expression, where we found statistically significant increases across all stages (117.8 ± 3.25%, 143 ± 4.23%, and 126.7 ± 4.02% for 24 h, 5 d, and end-stage, respectively, *p* < 0.001). Figure 3C shows mean LC3 expression; here, we found a significant increase after 24 h rotenone (118.2 ± 3.07%, *p* < 0.001), a significantly decreased expression after 5 d rotenone (78.16 ± 1.87%, *p* < 0.001) and in end-stage rats (88.33 ± 2.17%, *p* < 0.05). Analysis of OPTN puncta (Figure 3D) exhibited increased mean puncta per cel l across all stages; 67.77 ± 2.5, 91.9 ± 3.16, 120 ± 4.4, and 126.2 ± 4.3 (*p* < 0.001), for control, 24 h, 5 d, and end-stage, respectively. Mean LC3 puncta were increased across all stages, but only reached significance after 24 h rotenone (50.9 ± 2.1, *p* < 0.001 for 24 h; 42.8 ± 2.4, and 38.6 ± 1.5 for 5 d and end-stage, respectively) when compared to control (35.6 ± 1.2). Colocalized puncta per cell exhibited no differences after 24 h (23.5 ± 1.0), but significantly reduced number of puncta at 5 d and end-stage (17.3 ± 1.2 and 16.8 ± 1.1, respectively) when compared to control (22.5 ± 1.0; Figures 3E,F). We then considered changes in the percent of OPTN or LC3 puncta that were colocalized by PD stage. Results show decreased percent of OPTN puncta and LC3 puncta were found across all stages when compared to control; 40.8 ± 2.3%, 28.2 ± 1.3% (*p* < 0.01), 14.3 ± 0.8% (*p* < 0.001) and 13.9 ± 0.9% (*p* < 0.001) for OPTN, 65.8 ± 2.2%, 48.3 ± 1.7%, 41.7 ± 1.9%, and 43.7 ± 2.2% for LC3 (*p* < 0.001 for all), respectively (**Figures 6C,D**).

### Locus coeruleus (LC)

**Figure 4:**
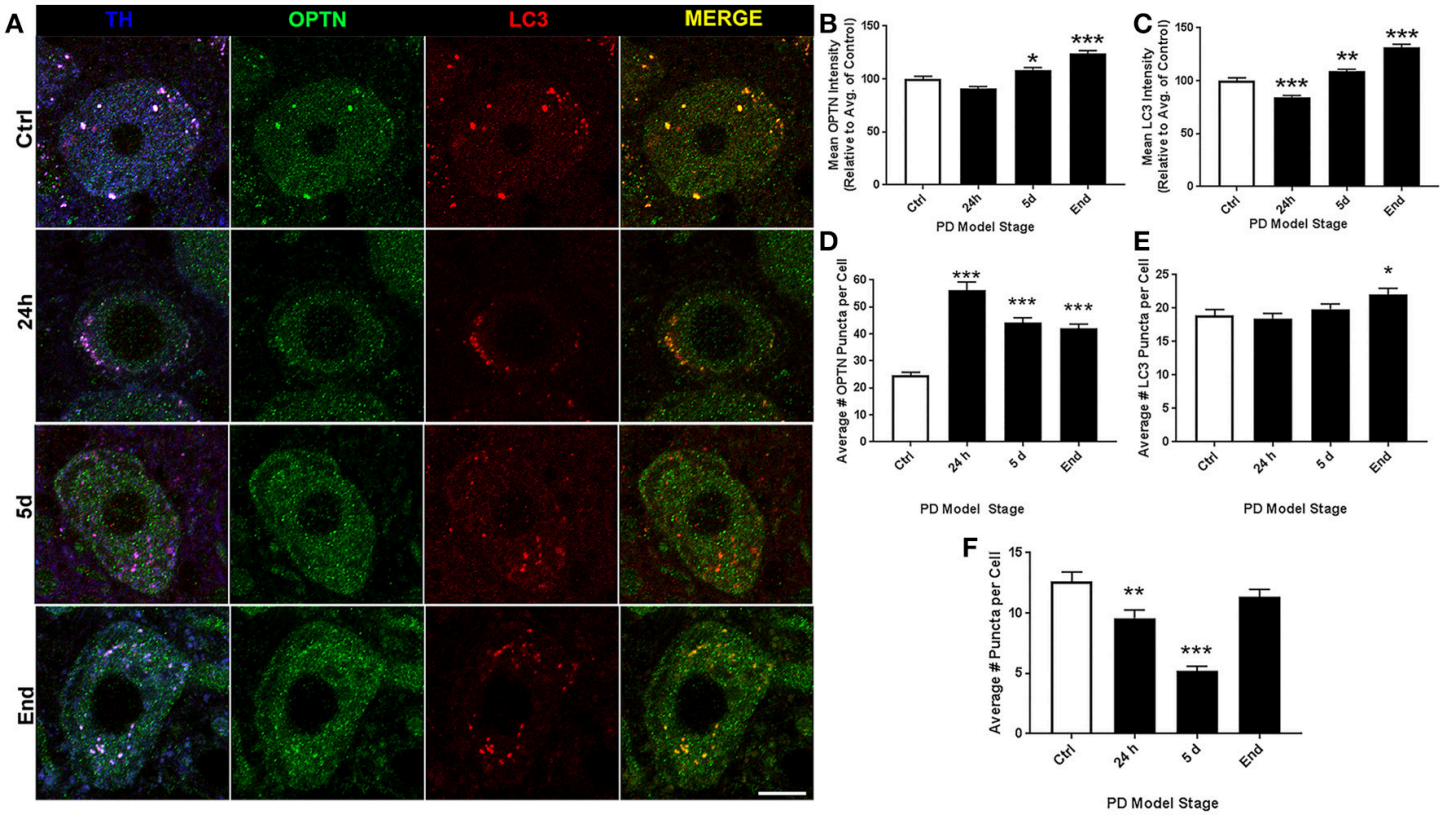
LC altered expression and puncta formation of OPTN and LC3 across PD stages. **(A)** Representative images of coerular dopaminergic neurons co-stained with OPTN, LC3, and TH from animals treated as control(top), 24 h (second from top), 5 d (second from bottom), or end-stage (bottom); ChAT in blue, OPTN in green, LC3 in red (scale bar = 10 μm). **(B,C)** Quantitative analysis of OPTN and LC3 expression, respectively, measured by relative intensity of total cellular expression (relative to control); **(D–F)** mean number of puncta per neuron for OPTN, LC3 or colocalized puncta, respectively, across stages; data are presented as mean ± SEM; ^*^p < 0.05, ^**^p < 0.01, ^***^p < 0.001 from control, Dunn's multiple comparison test after significant Kruskal-Wallis test.

Representative images of LC staining are shown in Figure 4A. For ROI analyses we evaluated 108, 123, 149, and 140 neurons for control, 24 h, 5 d, and end-stage rats, respectively. Figure 4B shows mean OPTN expression, where we observed a trend for increasing OPTN expression after rotenone; 91.18 ± 1.6%, 108.65 ± 2.27% (*p* < 0.05), and 124.19 ± 2.65% (*p* < 0.001) in 24 h, 5 d, and end-stage models, respectively. Figure 4C shows mean LC3 expression; here, we found an initial significant decrease after 24 h rotenone (84.64 ± 1.52%, *p* < 0.001), and significantly increased expression after 5 d rotenone (108.91 ± 1.97%, *p* < 0.01) and in end-stage rats (131.61 ± 2.98%, *p* < 0.001). Analysis of OPTN puncta (Figure 4D) exhibited increased mean puncta per cell across all PD model stages, but gradually decreasing mean puncta per cell with stage; 25 ± 1.1, 56 ± 3.0, 44 ± 1.7, and 42 ± 1.5 (*p* < 0.001 for all PD model stages) for control, 24 h, 5 d, and end-stage, respectively. Figure 4E shows mean LC3 puncta per cell exhibited little to no change in preclinical PD model stages when compared to control (19 ± 0.9, 18 ± 0.8, 20 ± 0.8 puncta per cell for control, 24 h, and 5 d), but showed a statistically significant increase in the end-stage PD model (22 ± 0.9 puncta per cell, *p* < *0.05*). Figure 4F shows mean colocalized puncta per cell; here, we observed a trend for decreasing mean colocalized puncta per cell in the preclinical PD stages but a return to control levels in the end-stage model. Mean colocalized puncta were 13 ± 0.8, 10 ± 0.7 (*p* < 0.01), 5 ± 0.4 (*p* < 0.001), and 11 ± 0.6 for control, 24 h, 5 d, and end-stage, respectively. We then considered changes in the percent of OPTN or LC3 puncta that were colocalized by PD stage. Results show decreased percent of OPTN puncta and LC3 puncta across all stages when compared to control; 40.8 ± 2.3%, 25.1 ± 1.7% (*p* < 0.001), 12.6 ± 0.9% (*p* < 0.001) and 28.3 ± 2.2% (*p* < 0.01) for OPTN, 65.8 ± 2.1%, 51.7 ± 1.9%, 25.9 ± 1.4% and 48.4 ± 2.0% for LC3 (*p* < 0.001 for all), respectively (**Figures 6E,F**).

### Pedunculopontine tegmental nucleus (PTg)

**Figure 5:**
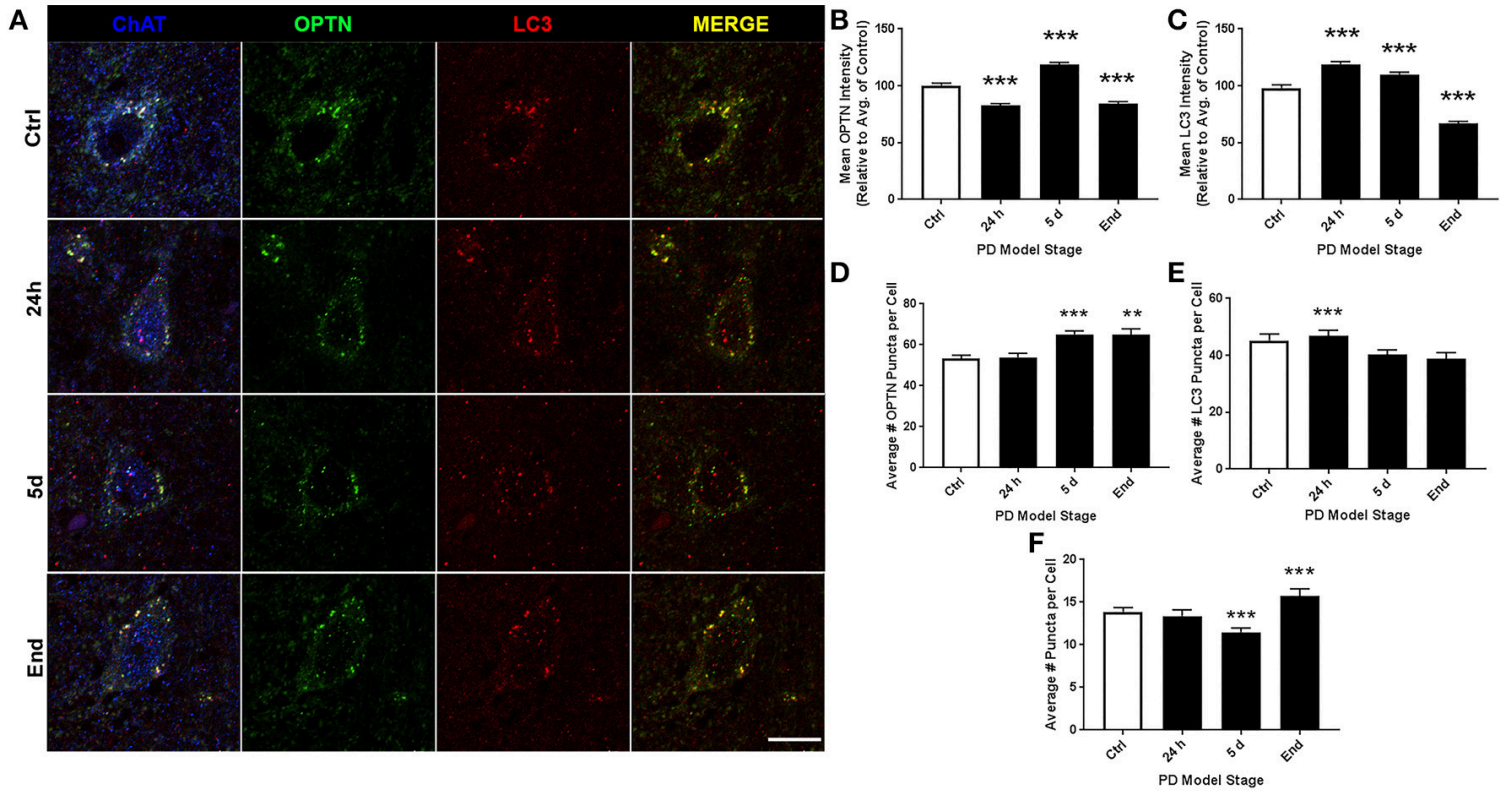
PTg altered expression and puncta formation of OPTN and LC3 across PD stages. **(A)** Representative images of pontotegmental cholinergic neurons co-stained with OPTN, LC3, and TPH from animals treated as control(top), 24 h (second from top), 5 d (second from bottom), or end-stage (bottom); ChAT in blue, OPTN in green, LC3 in red (scale bar = 10 μm). **(B,C)** Quantitative analysis of OPTN and LC3 expression, respectively, measured by relative intensity of total cellular expression (relative to control); **(D–F)** mean number of puncta per neuron for OPTN, LC3 or colocalized puncta, respectively, across stages; data are presented as mean ± SEM; ^**^p < 0.01, ^***^p < 0.001 from control, Dunn's multiple comparison test after significant Kruskal-Wallis test.

**Figure 6:**
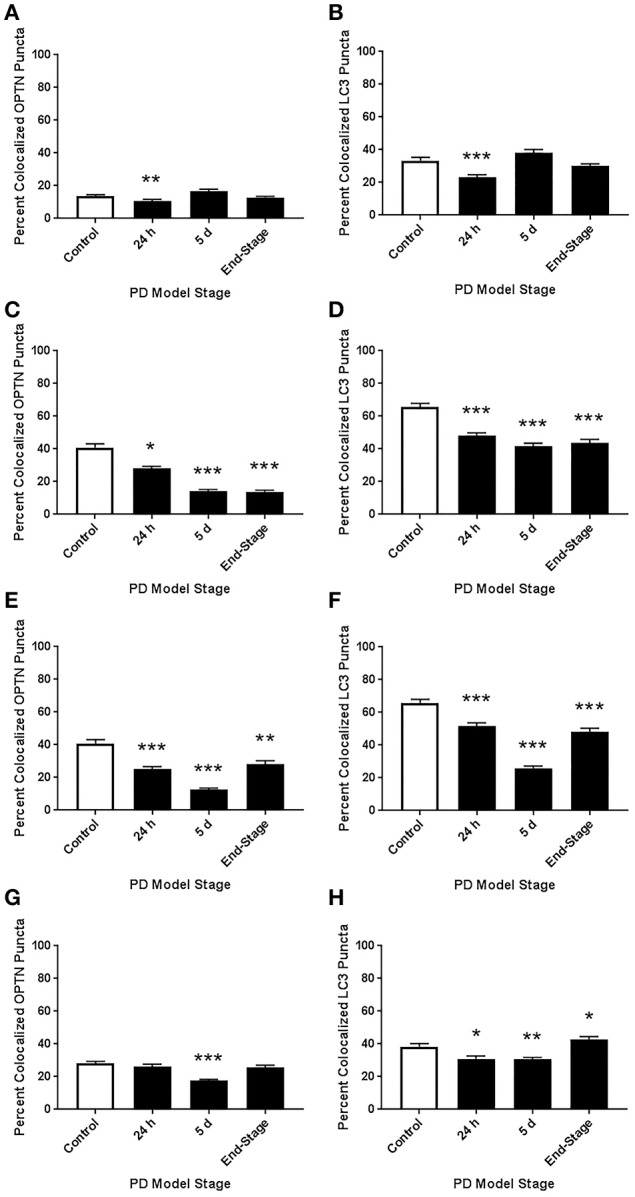
Altered colocalization of OPTN and LC3 across brainstem regions and PD stages. Here, we assessed changes in the colocalization of OPTN and LC3 in each region across all PD stages with our rotenone model. We quantified changes in the percent of OPTN puncta that were colocalized and changes in the percent of LC3 puncta that were colocalized in 10N **(A,B)**, RMg **(C,D)**, LC **(E,F)**, and PTg **(G,H)**. ^*^p < 0.05, ^**^p < 0.01, ^***^p < 0.001 from control, Dunn's multiple comparison test after significant Kruskal-Wallis test.

Representative images of PTg staining are shown in Figure 5A. For ROI analyses we evaluated 162, 121, 161, and 97 neurons for control, 24 h, 5 d, and end-stage rats, respectively. Figure 5B shows mean OPTN expression, where we observed significantly decreased OPTN expression after rotenone; 82.56 ± 1.8% (*p* < 0.001) and 84.37 ± 1.63% (*p* < 0.001) respectively; while OPTN expression was increased after 5 d rotenone (118.6 ± 2.15%, *p* < 0.001). Figure 5C shows mean LC3 expression; we found an initial significant increase after 24 h and 5d rotenone (119.1 ±2.26% and 110.1 ± 1.84%, *p* < 0.001, respectively) and significantly decreased expression in end-stage rats (66.91 ± 1.83%, *p* < 0.001). Analysis of OPTN puncta (Figure 5D) exhibited increased mean puncta per cell after 5 d rotenone and end-stage rats; 53.3 ± 1.6, 53.9 ± 2.0, 65.2 ± 1.7, and 64.9 ± 2.9 (*p* < 0.001) for control, 24 h, 5 d, and end-stage, respectively. Figure 5E shows mean LC3 puncta per cell exhibited significantly increased LC3 puncta after 24 h rotenone when compared to control (33.6 ± 1.2 and 50.9 ± 2.1 puncta per cell in control and 24 h, respectively; *p* < 0.001). Slightly increased values were observed in 5 d and end-stage PD models when compared to control, but were not statistically significant (42.8 ± 2.4 and 38.6 ± 1.5, respectively). Figure 5F shows mean colocalized puncta per cell; here, we observed significantly reduced mean colocalized puncta per cell after 5 d rotenone and in end-stage rats. Mean colocalized puncta were 22.5 ± 1.0, 23.5 ± 1.1, 17.3 ± 1.2 (*p* < 0.001), and 16.8 ± 1.0 (*p* < 0.001) for control, 24 h, 5 d, and end-stage, respectively. We then considered changes in the percent of OPTN or LC3 puncta that were colocalized by PD stage. Results show decreased percent of OPTN puncta only after 5 d rotenone when compared to control: 28.3 ± 1.1% and 17.8 ± 0.7% (*p* < 0.001), respectively. Results show decreased percent of LC3 puncta colocalized after 24h and 5 d rotenone, but increased at end-stage: 38.5 ± 1.8%, 30.9 ± 1.8% (*p* < 0.05), 30.5 ± 1.3% (*p* < 0.01) and 42.8 ± 1.7% (*p* < 0.05) for LC3, respectively (Figures 6G,H).

### Beclin-1 and alpha-synuclein expression in SNpc

**Figure 7:**
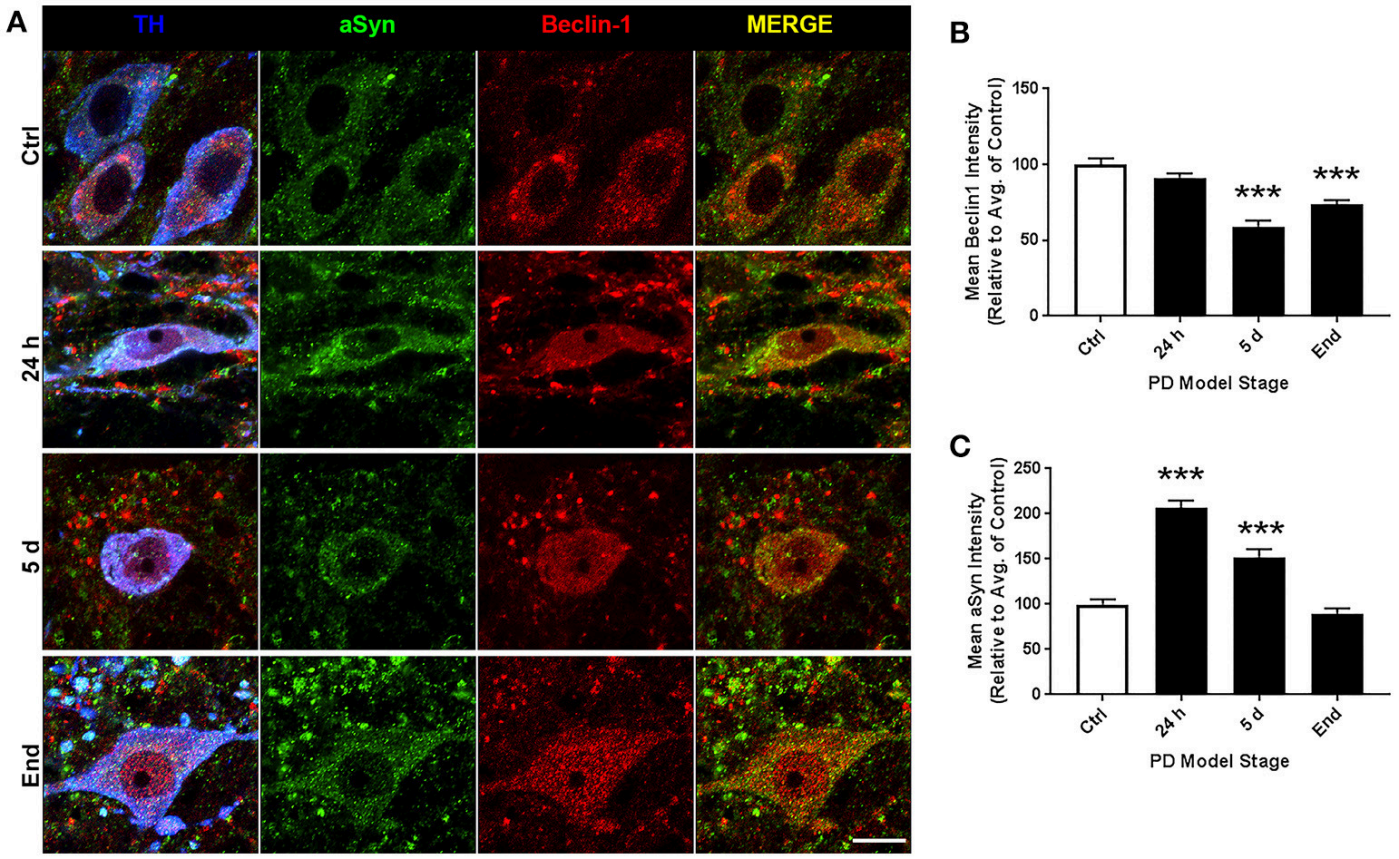
SN altered expression of Beclin-1 and alpha-synuclein across PD stages. **(A)** Representative images of nigral dopaminergic neurons co-stained with alpha-synuclein, beclin-1, and TH from animals treated as control(top), 24 h (second from top), 5 d (second from bottom), or end-stage (bottom); TH in blue, alpha-synuclein in green, beclin-1 in red (scale bar = 10 um). **(B,C)** Quantitative analysis of beclin-1 and alpha-synuclein expression, respectively, measured by relative intensity of total cellular expression (relative to control); data are presented as mean ± SEM; ^***^p < 0.001 from control, Dunn's multiple comparison test after significant Kruskal-Wallis test.

Representative images of SNpc staining are shown in Figure 7A. For ROI analyses we evaluated 158, 193, 99, and 160 neurons for control, 24 h, 5 d, and end-stage rats, respectively. Figure 7B shows mean beclin-1 expression, where we observed significantly decreased beclin-1 expression after 5 d rotenone and in end-stage rats; 59.16 ± 3.93% (*p* < 0.001) and 73.68 ± 2.85% (*p* < 0.001) respectively. Figure 7C shows mean alpha-synuclein expression; here, we found an initial significant increase after 24 h and 5d rotenone (206.4 ± 8.5% and 152.3 ± 8.73%, *p* < 0.001, respectively), and slightly decreased expression in end-stage rats (89.8 ± 5.49%, *p* < 0.001).

### Basal autophagic activity in brainstem regions

**Figure 8:**
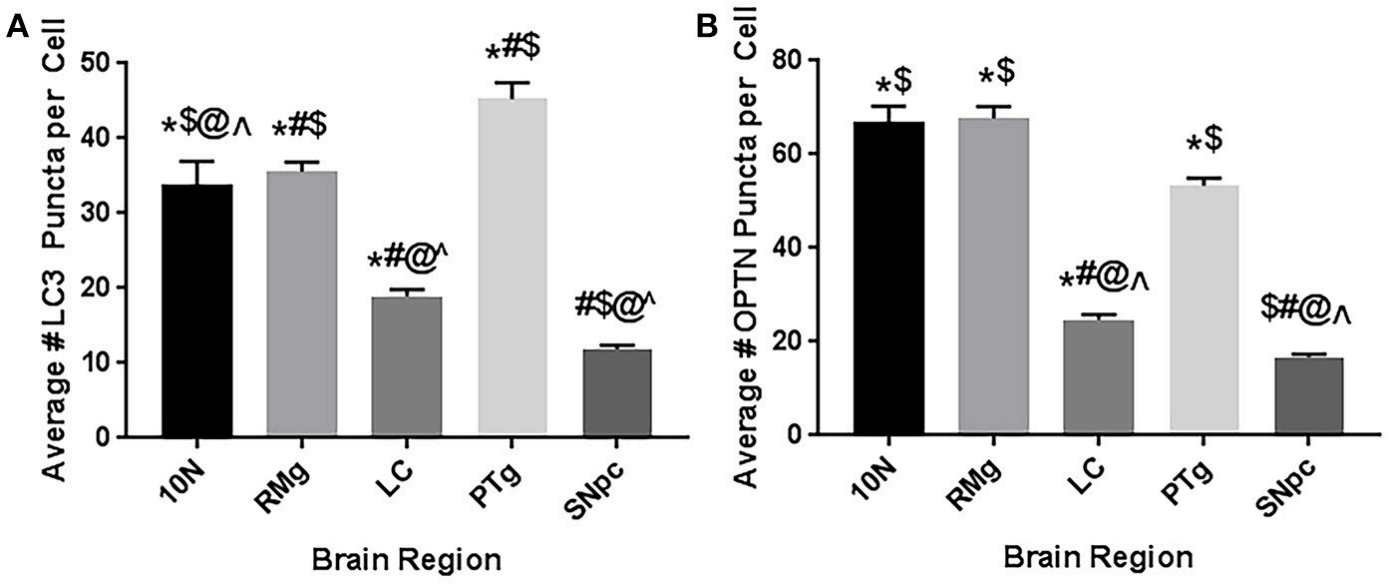
Basal autophagic activity in brainstem regions implicated in Braak's hypothesis. Data represents the mean number of **(A)** LC3 puncta (i.e., autophagosomes) or **(B)** OPTN puncta per cell in 10N, RMg, LC, PTg, and SNpc. Data suggest the dopaminergic neurons (LC and SNpc) exhibit less basal autophagic activity than other regions. ^*^p < 0.05 vs. SNpc; ^#^*p* < 0.05 vs. 10N; ^$^*p* < 0.05 vs. LC; ^@^*p* < 0.05 vs. RMg; ∧*p* < 0.05 vs. PTg.

Finally, we compared the mean OPTN and LC3 puncta in control animals across all brainstem regions considered here (Figure 8). We also considered data from our previous report to compare basal autophagic activity to SNpc as well. Our data suggest the dopaminergic neurons in LC and SNpc exhibit significantly lower autophagic activity than other regions. Average numbers of LC3 and OPTN puncta were significantly lower when compared to 10N, RMg, or PTg. Furthermore, average number of OPTN and LC3 puncta in dopaminergic neurons of SNpc were even lower than those of LC.

## Discussion

Autophagy disruptions have been repeatedly implicated in the pathogenesis of PD and other neurodegenerative diseases. However, the temporal development of autophagic changes is poorly characterized with respect to phenotype onset. Further, there is a lack of data on how brain regions outside the substantia nigra may be affected. In this study, we considered OPTN's autophagic role in four extranigral regions during PD pathogenesis by analyzing the expression and interaction of OPTN and LC3 using a rat rotenone model of PD. Collectively, we show that autophagy is affected in multiple brain nuclei important in PD, and that changes in these regions precede a lesion to the nigrostriatal dopamine system. Taking these data together, we can assess impairments to autophagy that may result in autophagic stress. While autophagy is ideally assessed dynamically (e.g., live-cell imaging), our approach to assess multiple endpoints and assessing populations of neurons improves our ability to assess its activity at a single time point. Finally, our data suggest dopaminergic neurons exhibit lowered basal autophagic activity, and may be limited in their autophagic capacity—especially those in the SNpc (see Figure 8). Such a limitation may render these neurons prone to cellular stress and neurodegeneration when autophagy is required for neuroprotection.

In this study, we consider the role of OPTN in autophagy as we believe it is the autophagy cargo adaptor most likely to contribute to PD. Thus far only two studies have considered OPTN in PD; a paper from Osawa and colleagues who immunostained for OPTN in patients with PD and our previous report considering OPTN expression, puncta, and colocalization with LC3 in the same rat rotenone model we used here (Osawa et al., 2011; Wise and Cannon, 2016). The report by Osawa et al. is very limited for its investigation of OPTN in PD; only two high magnification images are shown of what the authors report as an OPTN-positive Lewy body and Lewy neurite, but no comparison to a control patient was provided and no validation of the criteria for Lewy bodies and neurites (e.g., alpha-synuclein immunostain; Osawa et al., 2011). OPTN was previously demonstrated to be an essential cargo adaptor for mitophagy, and mitophagy is critical for clearing dysfunctional mitochondria from neuronal populations (Wong and Holzbaur, 2014). Here, we used rotenone as a PD model; rotenone is a mitochondrial complex I inhibitor, and is known to induce mitophagy in neuronal populations (Pan et al., 2009; Gao et al., 2012; Chu et al., 2013). Thus, it is very likely that the changes we see in OPTN expression and puncta formation are linked to mitochondrial dysfunction and mitophagy activity. Furthermore, OPTN is the most likely candidate because from a recent genome-wide association study, only the OPTN^M98K^ mutation was found to significantly increase risk of PD when all known autophagy cargo adaptors were considered (Lill et al., 2012). Importantly, we show OPTN is expressed in multiple extranigral regions that are implicated in Braak's hypothesis of PD progression (10N, RMg, LC, PTg). In each region, OPTN expression was more robust than surrounding nuclei, further implying a potential role for OPTN in PD pathogenesis (Braak and Braak, 2000; Braak et al., 2003).

Autophagy is a complex process that requires considerable cellular resources to execute; e.g., cell signaling for induction, recruitment of membranes from other organelles, complex signaling to identify and recruit cargo and to encourage autophagosome-lysosome fusion (Behrends et al., 2010; Glick et al., 2010; Damme et al., 2015). Despite the many complexities required to complete autophagy from induction to cargo degradation, there appear to be relatively few pathological endpoints that can be observed *in vivo*: autophagy is induced but unaffected (exhibiting increase LC3 expression and puncta, with decreased OPTN), impaired induction (reducing LC3 expression and number of autophagosomes), impaired cargo recruitment (e.g., OPTN-LC3 binding), or impaired autophagosome-lysosome fusion (resulting in increased LC3 expression and number of autophagosomes). Another potential impairment in autophagy lies in the activity of cargo adaptors (e.g., OPTN, p62, NBR1), though this is much more difficult to detect *in vivo* as many of these proteins require post-translational modifications to encourage binding to cargo or LC3 (Rogov et al., 2014). Based on this literature, we present a means of detecting impairment of cargo adaptor-LC3 interaction by quantifying the percent of LC3 or cargo adaptor puncta that are colocalized. With this method of analysis, we can evaluate potential impairments to autophagy induction as: (1) a decrease in the percent of colocalized OPTN puncta, without changes in colocalized LC3 puncta as indication of fewer autophagosomes to bind OPTN puncta, but interaction is unchanged; (2) impaired OPTN-LC3 interaction as decreased percent of colocalized puncta for both OPTN and LC3; or (3) impaired autophagosome-lysosome fusion as an increase in both. We can also evaluate changes in OPTN's activity as a cargo adaptor as: (1) no change or a decrease in the percent of colocalized OPTN puncta coinciding with increased percent of LC3 colocalized puncta; or (2) the availability of autophagosomes (i.e., autophagosome depletion) as no change or an increase in the percent of LC3 puncta coinciding with a decrease in the percent of colocalized OPTN puncta.

PD is well characterized as a systemic disorder, with extensive pathology beyond that in nigral dopaminergic neurons. Despite this, the vast majority of PD research still focuses on nigral pathology, dopaminergic dysfunction, or dopaminergic neuroprotection. Previous studies have shown histological evidence of alpha-synuclein aggregation in dopamine neurons of the substantia nigra of rats treated with the rotenone dose used in this study (Betarbet et al., 2000; Cannon et al., 2009). Of note, these studies showed qualitative evidence of the formation of Lewy body-like aggregates, whereas, here we conducted whole-cell ROI analysis. Interestingly, we found whole-cell alpha-synuclein levels to increase acutely and then return near baseline (Figure 7C). Indeed, while whole-cell synuclein levels decrease during the course of treatment, aggregate formation is also apparent in our data (Figure 7A). This result may be indicative of dynamic redistribution during aggregate formation that has been described in cell culture experiments for PD and other neurodegenerative diseases (Arrasate et al., 2004; Opazo et al., 2008). While the etiology of PD remains elusive, Braak et al. have published a substantial amount of work showing progression of LBP through distinct synaptic tracts in the PD brain, but how LBP progresses from one region to another remains unclear. Given that large protein aggregates are normally cleared via autophagy, we proposed autophagic dysfunction might follow a similar pattern and precede LBP. Intriguingly, we observed decreasing beclin-1 expression coinciding with alpha-synuclein expression redistribution from cytosolic expression to more punctate expression (Figure 7). Indeed, beclin-1 has been shown to aid in autophagic clearance of alpha-synuclein aggregates and its dysfunction may have a pivotal role in autophagic dysfunction (Spencer et al., 2009). Considering the autophagic pathway (see Figure 1), we defined three possible outcomes of impairment that could be detected *in vivo* using our immunohistochemical staining: (1) impaired induction, (2) impaired OPTN-LC3 binding, and (3) impaired autophagosome-lysosome fusion. We also considered the possibilities of OPTN failing to serve as a cargo adaptor protein and of depleted autophagosome pool. Interestingly, our results suggest different types of impairment in different regions (see Table 2). Most of our data suggest impairments in OPTN-LC3 binding (perhaps due to impaired phosphorylation of OPTN by TBK1) or impaired autophagosome-lysosome fusion. Specifically, results from the 10N suggest early disruptions (after 24 h rotenone) in LC3-OPTN binding (evidenced by significantly decreased colocalized puncta in Figure 2F and significantly decreased percent of colocalized puncta in Figures 6A,B), and later impairment of autophagy induction (based on decreased number of LC3 puncta in Figure 2E). The RMg largely exhibited pathology that suggested impaired LC3-OPTN interaction and autophagosome-lysosome fusion across all stages (based on consistently decreased percent of colocalized puncta seen in Figures 6C,D), and likely exhaustion of the autophagosome pool at end-stage PD (especially when considering the decreasing number of LC3 puncta coinciding with increasing OPTN puncta in Figures 3D,E). Data from LC suggest autophagy induction is initially impaired (based on 24 h expression data in Figures 4B,C) with impaired LC3-OPTN interactions (see Figure 4F), but then shifts entirely to impaired binding between LC3 and OPTN (based on puncta analyses in Figures 4F, 6E,F). There also seems to be potential impairment of autophagosome-lysosome fusion after 5 d rotenone, especially when considering the accumulation of LC3 and colocalized puncta (Figure 4F) and percent of colocalized puncta (Figures 6E,F), where we see increases in these values between 5 d and end-stage, reversing the apparent trend of decreasing values between control, 24 h, and 5d. Data from the PTg collectively showed the least amount of autophagic impairments. Importantly, we see especially little pathology after 24 h rotenone, where we saw signs of autophagic dysfunction in lower brainstem regions. We began to see signs of autophagic dysfunction in the PTg after 5 d rotenone, where there appears to be impaired LC3-OPTN binding (significantly decreased number of colocalized puncta in Figure 5F and percent of colocalized LC3 and OPTN puncta in Figures 6G,H) and/or autophagosome-lysosome fusion (significantly increased colocalized puncta in Figure 5F). Alternatively, these data could indicate autophagosome depletion, where we see decreasing number of autophagosomes with increasing PD severity (Figure 5E) and significantly increased percent of colocalized LC3 puncta but no change in percent of colocalized OPTN puncta in end-stage animals (Figures 6G,H). While these data do not indicate if autophagic dysfunction is occurring ahead of LB pathology progression, the data do suggest a similar pattern—with lower brainstem regions (10N, RMg, LC) exhibiting signs of autophagic dysfunction earlier than the PTg which is the brainstem region highest in the brain and last affected of the regions considered in this study.

**Table 2:** Overall summary of autophagic changes: *Results for OPTN and LC3*.

	**Braak region:**	**10N**			**RMg**			**LC**			**PTg**		
	**PD model stage*:***	**24 h**	**5d**	**End**	**24 h**	**5d**	**End**	**24 h**	**5d**	**End**	**24 h**	**5d**	**End**
	**Expected outcome**												
Autophagy Induced, flux unimpaired	Expression: Increased LC3, OPTN decreased or no change	**X**									**X**		
	# Puncta: LC3 increased, OPTN decreased or no change in colocalized	**/**									**X**		
	% Colocalized Puncta: No changes												
(1) Impaired Induction	Expression: Decreased LC3, OPTN decreased or no change							**X**					**X**
	# Puncta: OPTN increased or no change, LC3 and colocalized decreased	**/**	**X**									**/**	
	% Colocalized Puncta: % colocalized OPTN decreased, no change in % colocalized LC3												
(2) Impaired OPTN-LC3 Binding	Expression: Decreased LC3, increased OPTN												
	# Puncta: OPTN increased, LC3 decreased or no change, colocalized decreased			**/**		**/**		**X**	**X**	**/**		**/**	
	% Colocalized Puncta: Decreased LC3 and OPTN	**X**			**X**	**X**	**X**	**X**	**X**	**X**	**/**	**X**	
(3) Impaired Autophagosome-Lysosome Fusion	Expression: Increased LC3 and OPTN		**X**	**X**	**X**	**/**	**/**		**X**	**X**		**X**	
	# Puncta: All increased (colocalized increased substantially)				**X**	**/**				**/**			**/**
	% Colocalized Puncta: Increased LC3 and OPTN												
OPTN Failure or Exhaustion	Expression: Decreased OPTN, unknown effect on LC3	**/**									**/**		
	# Puncta: Decreased OPTN, unknown effect on LC3, decreased colocalized	**/**											
	% Colocalized Puncta: Decreased LC3, increased OPTN												
Autophagosomeexhaustion	Expression: Decreased LC3, increased OPTN					**/**	**/**						
	# Puncta: Increased OPTN, decreased LC3 and colocalized			**/**			**X**					**/**	**/**
	% Colocalized Puncta: Increased LC3, decreased OPTN												**/**

*We identified four possible endpoints to assess autophagy impairments in vivo when considering our data points: autophagy is induced but unaffected (i.e., autophagy is functioning normally), autophagy induction is impaired, OPTN-LC3 binding is impaired, or autophagosome-lysosome fusion are impaired. “X” indicates all criteria were met, whereas “/” indicates criteria were partially met*.

Given that the nigral dopaminergic neurons exhibit the most severe deterioration in PD, much research has considered what makes this population selectively vulnerable. Collectively, the literature points to limitations in the energetic capacity of these neurons. Nigral dopamine neurons are characterized by dense and extensive dendritic processes, with each neuron exhibiting thousands of synaptic connections (Matsuda et al., 2009). This, combined with poorly myelinated processes, puts a significant amount of stress on the energy demand of the neurons (Braak et al., 2003; Matsuda et al., 2009). Compounding these high-energy demands, dopaminergic neurons of the SNpc were reported to have relatively low mitochondrial populations and energetic capacities per neuron (Pacelli et al., 2015). Our results suggest yet another reason why these neurons might be selectively vulnerable; when we consider the mean number of LC3 puncta per neuron in control animals across all the regions considered, SNpc dopaminergic neurons exhibited the fewest, while the cholinergic neurons of the PTg exhibited the highest number of LC3 puncta. If these neurons exhibit limited autophagic capacity, they would be severely limited in their ability to clear pathogenic protein aggregates such as Lewy bodies and dysfunctional mitochondria that leak reactive oxygen species. Such a limitation could create a negative feedback loop, with increased oxidative stress from dysfunctional mitochondria impairing the already limited autophagic capacity of these neurons and thus prolonging the damage incurred. This hypothesis is further supported by Sato et al. who reported their results from mice with selective autophagy-deficiency in dopaminergic neurons in mice. In their report, these autophagy-deficient mice exhibited behavior and pathology in LC and SNpc akin to other PD models (Sato et al., 2018).

Assessing autophagic dysfunction *in vivo* has been extremely limited due to its dynamic and microscopic process (Klionsky et al., 2016). *In vivo* assessment of autophagy through immunohistochemistry is an area of autophagy research that is severely limited and lacking standardized analyses (Klionsky et al., 2016). Here, we presented a new manner of considering autophagic dysfunction *in vivo* using a multivariate analysis approach: (1) quantifying expression of LC3 and OPTN (a known autophagy cargo-adaptor), (2) quantifying puncta formation of each (also considering colocalized puncta), and (3) by assessing changes to the percent of total LC3 and OPTN puncta that are colocalized. This novel approach to *in vivo* autophagy analysis enables the evaluation of several critical steps in autophagy flux. Here, we use this approach to asses changes in autophagy induction, interaction between LC3 and OPTN (an autophagy cargo adaptor), autophagosome-lysosome fusion, and exhaustion or depletion of OPTN serving a cargo adaptor function or availability of autophagosomes. Given that autophagy is a complex process, the best approach to analyze its function or dysfunction utilizes live-cell imaging and pharmacological inhibition of specific junctions (e.g., induction or autophagosome-lysosome fusion). To address the challenge of autophagy changes over time and to assess autophagic activity changes over the course of PD progression, we considered these endpoints using a thoroughly characterized dosing scheme for the PD neurotoxin rotenone. Our results demonstrate variable types of impairment in extranigral regions implicated in preclinical PD, which supports our hypothesis that autophagic dysfunction may precede the spread of Lewy bodies through the PD brain. Further investigation is needed to understand the complexity of autophagic dysfunction in these regions and how it contributes to PD progression. Importantly, if autophagic dysfunction precedes LBP, it may become an important target for future PD therapeutics to help slow the progression of the disease.

## Author contributions

JW Designed, performed, and analyzed experiments; wrote the 1st draft, and subsequent drafts. CP Performed and analyzed experiments. JA Performed and analyzed experiments. JC Responsible for all aspects of the research design, execution and interpretation; review and refinement of initial and subsequent manuscript drafts. JC also funded the work through internal and extramural awards.

### Conflict of interest statement

The authors declare that the research was conducted in the absence of any commercial or financial relationships that could be construed as a potential conflict of interest.
